# Case report of recurrent acute appendicitis in a residual tip

**DOI:** 10.1186/1757-1626-3-14

**Published:** 2010-01-09

**Authors:** Donal P O'Leary, Eddie Myers, Joe Coyle, Ian Wilson

**Affiliations:** 1 Department of Surgery, Saint Luke's Hospital, Kilkenny, Ireland

## Abstract

**Introduction:**

Residual appendicitis involving the stump of the appendix has been well described in the literature in the past.

**Case report:**

We report the case of a 43 year old male with acute onset of abdominal pain who had undergone an appendicectomy ten years previously. Ultrasound revealed the presence of an inflamed tubular structure. Subsequent laparotomy and histology confirmed that this structure was an inflamed residual appendiceal tip.

**Conclusion:**

Residual tip appendicitis has not been reported in the literature previously and should be considered in the differential diagnosis of localised peritonitis in a patient with a history of a previous open appendicectomy.

## Introduction

Acute appendicitis is a common surgical emergency requiring intervention. The lifetime risk of developing appendicitis is approximately seven per cent [1]. Despite advances in both minimally invasive surgery and radiology, it's accurate diagnosis remains challenging in some instances [2]. This is especially the case following a previous appendicectomy, where inflammation of residual appendiceal tissue is a possibility.

## Case Report

**Figure 1:**
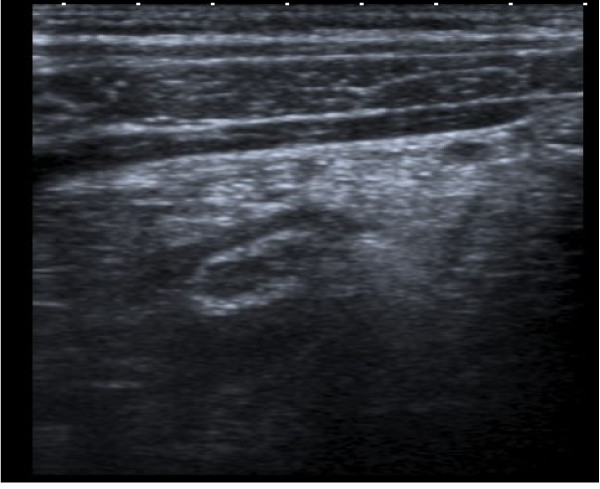
**Ultrasound showing an inflamed tubular structure**.

We report the case of a 43 year old male who presented with sudden onset of severe abdominal pain. His symptoms were consistent with acute cholecystitis. He was unable to speak English and communicated via an interpreter. On examination a Battle's incision was noted. He claimed that this was due to a previous cholecystectomy completed ten years previous in his home country. He demonstrated signs of localised peritonitis in the right upper quadrant. Inflammatory markers were elevated. A clinical diagnosis of acute cholecystitis was made and broad spectrum antibiotics commenced. An ultrasound was performed and this confirmed the presence of a non-inflamed gallbladder which did not contain gallstones. However, it did reveal an inflamed tubular, non-compressible, non-peristalting, blind-ending structure intimately related to the liver suggesting sub-hepatic appendicitis (Figure 1).

Laparoscopy revealed dense adhesions throughout the abdominal cavity and the presence of purulent intra-abdominal fluid. We proceeded to a laparotomy through a midline incision. Following extensive adhesiolysis the caecum was identified, however only a non-inflamed appendiceal stump remained. Further dissection revealed evidence of pus in the right sub-hepatic space. The pus was originating from a tubular structure which was adherent to the lateral abdominal wall. An intra-operative diagnosis of residual tip appendicitis was made. The tubular structure, measuring 2.5 cms in length, was removed and was confirmed histologically as appendicitis with evidence of acute inflammatory changes. The patient made an uneventful recovery and was discharged home six days post-operatively.

## Discussion

Appendicectomy is usually performed either via open or laparoscopic surgery. At open surgery the conventional incisions are of either the Gridiron or Lanz variety. A Battle's incision, which employs a vertical para-median incision with temporary retraction of the rectus muscle medially, was initially described in 1895 [3]. In the modern era it is rarely employed. In this setting with a battle's incision and limited clinical information, recurrent appendicitis formed part of the differential diagnosis, although the location of signs was atypical for stump appendicitis.

There are thirty seven cases of residual appendicitis in the English literature. The majority of these case reports involve stump appendicitis. Stump appendicitis remains, however, an under reported condition. It occurs when there is incomplete resection of the inflamed appendix. The reported interval between operations varies between two months and 50 years [4,5]. In our case of inflamed residual appendiceal tip, presentation was ten years post appendicectomy.

Residual appendicitis may be diagnosed using radiological imaging. Ultrasound imaging is useful and was diagnostic in this case. A pre-operative ultrasound, which was done primarily to confirm the presence of a gallbladder, visualised a sub-hepatic appendicitis. Computed tomography (CT) is also useful in diagnosing residual appendicitis. CT features of stump appendicitis are similar to those of acute appendicitis. They include pericaecal inflammatory changes, abscess formation, fluid in the right paracolic gutter, caecal wall thickening, and an ileocecal mass. Barium studies have also been reported as being diagnostic in diagnosing stump appendicitis [6]. There are also reported cases of stump appendicitis being diagnosed using colonoscopy [7].

The surgical treatment of residual appendicitis is most commonly reported as an open operation but cases have been successfully treated using laparoscopic intervention. This case required a laparotomy as there were dense adhesions from previous surgery and the exact source of purulent fluid was not evident laparoscopically. Residual appendicitis has been reported most frequently in cases of appendicitis treated with laparoscopic surgery. There is a high morbidity rate in this setting due to a combination of delayed diagnosis and a high rate of stump perforation [8]. The rate of perforation of stump appendicitis at the time of operation has been reported as sixty-eight per cent in one review of the literature [9].

## Conclusion

Residual tip appendicitis has not been reported in the literature previously and should be considered in the differential diagnosis of localised peritonitis in a patient with a history of a previous open appendicectomy.

## Consent

Written informed consent was obtained from the patient for publication of this case report and accompanying images. A copy of the written consent is available for review by the journal's Editor-in-Chief.

## Competing interests

The authors declare that they have no competing interests.

## Authors' contributions

EM - Assistant surgeon, JC - Radiologist who conducted ultrasound, IW - Consultant who operated on patient. All authors read and approved the final manuscript.
